# Structural basis for binding the TREX2 complex to nuclear pores, *GAL1* localisation and mRNA export

**DOI:** 10.1093/nar/gku252

**Published:** 2014-04-04

**Authors:** Divyang Jani, Eugene Valkov, Murray Stewart

**Affiliations:** MRC Laboratory of Molecular Biology, Francis Crick Avenue, Cambridge Biomedical Campus, Cambridge, CB2 0QH, UK

## Abstract

The conserved Sac3:Thp1:Sem1:Sus1:Cdc31 (TREX2) complex binds to nuclear pore complexes (NPCs) and, in addition to integrating mRNA nuclear export with preceding steps in the gene expression pathway, facilitates re-positioning of highly regulated actively transcribing genes (such as *GAL1*) to NPCs. Although TREX2 is thought to bind NPC protein Nup1, defining the precise role of this interaction has been frustrated by the complex pleiotropic phenotype exhibited by *nup1Δ* strains. To provide a structural framework for understanding the binding of TREX2 to NPCs and its function in the gene expression pathway, we have determined the structure of the Nup1:TREX2 interaction interface and used this information to engineer a Sac3 variant that impairs NPC binding while not compromising TREX2 assembly. This variant inhibited the NPC association of both de-repressed and activated *GAL1* and also produced mRNA export and growth defects. These results indicate that the TREX2:Nup1 interaction facilitates the efficient nuclear export of bulk mRNA together with the re-positioning of *GAL1* to NPCs that is required for transcriptional control that is mediated by removal of SUMO from repressors by NPC-bound Ulp1.

## INTRODUCTION

In addition to facilitating nucleocytoplasmic transport, nuclear pore complexes (NPCs) also orchestrate earlier steps of the gene expression pathway. In budding yeast, the TREX2 complex binds to NPCs and is thought to function in integrating the nuclear export of mature transcripts with earlier steps in the gene expression pathway, together with promoting genome stability through inhibiting R-loop formation (1–4). Many mutants in TREX2 components generate cellular growth defects and mRNA export defects that lead to nuclear accumulation of poly(A)+ mRNA and also frequently impair the binding of TREX2 to NPCs (reviewed by 3,5–6). Moreover, a subset of highly regulated genes (including *INO1, HXK1* and the *GAL7-GAL10-GAL1* cluster) become re-located to the nuclear envelope when their transcription is activated (3,7–11). In several cases this relocation has been shown to require both the SAGA (Spt-Ada-Gcn5 acetyltransferase) complex and TREX2 (7,12–13).

TREX2 is constructed from a Sac3 scaffold to which Thp1, Sem1, Cdc31 and two Sus1 chains bind (12,14–18). Sus1 and Cdc31 bind to the Sac3 centrin binding (CID) region (Sac3^CID^, residues 723–805) that has an unusually long α-helix about which the other three chains coil (17). Thp1 and Sem1 bind to the central region of Sac3 (Sac3M) where they generate two juxtaposed winged-helix domains that bind nucleic acid (18). At steady state, TREX2 is localized to NPCs, which is thought to be mediated primarily through an interaction between its CID region and the NPC protein (nucleoporin) Nup1 (14,16). Mutations that impair the binding of Sus1 or Cdc31 to Sac3^CID^ or that impair either the Thp1:Sac3 interaction or the binding of mRNA to this region, generate defects in both mRNA export and cell growth (17,18).

The NPC association of the *GAL* gene cluster contributes to its regulation that is mediated by both repression in the presence of glucose and activation in the presence of galactose. Glucose-induced repression is mediated by Mig1 in conjunction with the co-repressors Ssn6 and Tup1. De-repression occurs when glucose is replaced by either galactose or other carbon sources, such as raffinose, and is mediated by Ulp1 that desumoylates Ssn6 and Tup1 (19). Ulp1 is located at the NPC through an interaction with Mlp1 and Mlp2 and so locating the *GAL* cluster to the NPC when glucose is replaced by another carbon source facilitates de-repression by desumoylation of gene-bound Ssn6 and Tup1 (19). Conversely, it has been proposed that, under repressive conditions, inducible genes are located away from NPCs and so inaccessible to Ulp1, allowing sumoylation of chromatin-bound regulators to be maintained (19). Furthermore, galactose activates transcription by releasing the transcriptional activator Gal4 from its complex with Gal80 and several studies indicate that the NPC may facilitate gene-activator binding, suggesting that NPCs function in promoting transcription, facilitating relief of repression and promoting activation (19).

The relocation of activated, regulated genes such as *GAL1, HXK1* and *INO1* is thought to enhance the efficiency of transcription and the nuclear export of their transcripts, and thus the overall efficiency of gene expression, and has also been proposed to contribute to a form of epigenetic memory, albeit this may also result from residual components of the control of these pathways (3,7,8,10,19–21). The relocation of *GAL1* to the nuclear periphery following its activation is impaired by deletion of *SUS1*, *NUP1*, *SAC3* or components of the SAGA complex (3,7,22) and recent studies using q-PCR to measure *GAL1* transcript levels in *ada2Δ* (a SAGA component) or *nup1Δ* strains have suggested that the NPC association of *GAL1* may also ensure rapid repression following gene inactivation (22). Because deletion of *NUP1* generates complex pleiotropic phenotypes that depend on the yeast strain employed (23), it has been difficult to distinguish between phenotypic effects arising from changes in TREX2 composition, from those resulting from TREX2 localisation or from those associated with other areas of nucleocytoplasmic transport that involve Nup1, such as the kinetics of nuclear protein import complex disassembly (24,25).

We report here the crystal structure of the interface between TREX2 and Nup1 that shows that Nup1 residues 330–340 bind primarily to a cavity formed between the Sac3 helix (residues 757–777) and the Sus1 chain closest to Cdc31 (Sus1B) and show that Phe336^Nup1^ and Phe338^Nup1^ are crucial for this interaction. Although this interaction shares similarities with how nuclear transport factors bind to nucleoporin FG (phenylalanine-glycine) repeats, the TREX2:Nup1 interaction is structurally and functionally different. The interface structure was used to engineer a Sac3 variant in which binding to Nup1 was impaired *in vitro* but in which TREX2 assembly was not compromised. This variant impaired Sac3 localisation to NPCs *in vivo* and also reduced substantially the relocation of *GAL1* to the nuclear envelope upon either de-repression or activation. Moreover, this Sac3 variant generated cellular growth and mRNA export defects consistent with TREX2 increasing the efficiency of mRNA export in the gene expression pathway.

## MATERIALS AND METHODS

### Protein expression and purification

Nup1, Nup1 mutants, Nsp1 and Nup60 fragments were generated by polymerase chain reaction (PCR) from yeast genomic DNA (Novagen; Beston, UK) and cloned into pGEX6P-1 or pGEXTEV, a modified version of pGEX-4T-1 (GE Healthcare) in which the thrombin site has been replaced with a Tobacco Etch Virus (TEV) protease site. Sac3 fragments 753–805 (BC) and 723–805 (ABC), Cdc31 and Sus1 were cloned as described (17,26). Additional Sac3 fragments (residues 723–753 (A), residues 757–787 (B), residues 723–787 (AB) and residues 787–805 (C)) were generated by PCR and cloned into pGEXTEV. Sac3^CID^ domain mutants were created by overlap extension PCR and the resulting mutant Sac3 753–805 and 723–805 fragments cloned into pGEXTEV. All proteins were expressed in BL21 (DE3) CodonPlus RIL cells (Stratagene) at 20°C overnight following 1 mM IPTG induction. Sac3^CID^ domain sub-complexes were expressed and purified essentially as described (17). For Sac3^723^^–805^ and Sac3^753^^–805^ complexes, cells containing co-expressed Sac3:Cdc31 complex were mixed with cells containing Sus1 to excess (17). Cloning and purification of yeast Kap95 was as described (25).

For crystallography, cells containing GST-Nup1 fragments were lysed by high pressure cavitation (10–15k psi) in 50 mM Tris-HCl pH 8.0, 25% w/v sucrose, 1 mM EGTA, 1 mM PMSF and 2 mM DTT. Complete EDTA-free protease inhibitor mixture (Roche), DNase (Sigma) and further PMSF were added to the lysed cells, which were then incubated at 4°C for 30 min. Clarified cell lysate containing the GST-Nup1 fragment was bound to Glutathione Sepharose 4B resin (GE Healthcare) for 1 h. The resin was washed with 500 ml of phosphate buffered saline (PBS) supplemented with 1 mM DTT (PBS-DTT) and the Nup1 fragment then released by overnight digestion at 4°C with 100 μg of His-TEV protease (S219V mutant (27)). The fragment was further purified on a HiLoad Superdex75 26/60 Prep Grade column (GE Healthcare) in 20 mM Tris-HCl pH 8.0, 50 mM NaCl and 1 mM DTT. Because Nup1 fragments generally lacked Trp or Tyr, protein concentration was estimated by peptide bond absorption at 205, 215 and 225 nm.

### *In vitro* binding studies

The interaction between the Sac3^CID^ domain complex, sub-complexes, mutant variants and Kap95 with Nup1, Nup60 and Nsp1 were assayed using immobilized GST-Nup fusion proteins on Glutathione Sepharose 4B resin from clarified bacterial cell lysates. Resin-bound fragments were washed with PBS-DTT and purified Sac3^CID^ domain complex, or sub-complexes, mutant variants or Kap95 (as appropriate) added to 200 μl resin aliquots to a final concentration of 15.4 μM in a total reaction volume of 260 μl. After incubation at 4°C for 1 h, samples were washed with PBS-DTT and resin-bound material analysed by sodium dodecyl sulphate-polyacrylamide gel electrophoresis (SDS-PAGE) and Coomassie staining. No binding was observed to Glutathione S-transferrase (GST) alone.

Binding of Sus1 and Cdc31 to mutant Sac3^CID^ domain fragments was assayed by mixing clarified cell lysate containing co-expressed GST-Sac3^753–805^ and Cdc31 with clarified cell lysate containing Sus1 and incubated with Glutathione Sepharose 4B resin at 4°C for 1 h. The resin was washed with 50 mM Tris-HCl pH 8.0, 200 mM NaCl and 1 mM DTT and resin-bound material analysed by SDS-PAGE and Coomassie staining.

### Crystallography

A range of Nup1 fragments derived from Nup1^322–370^ were generated and each was mixed with Sac3^CID^ domain sub-complexes in a range of molar ratios and incubated on ice for 90 min prior to setting up crystallisation trials. Large ∼100 μm *P3_2_21* crystals were obtained by vapour diffusion when Nup1^322–355^ was mixed in a 6:1 molar ratio with Sac3^757–787^:Sus1B (15 mg/ml) in 2.2–2.35 M ammonium sulphate, 100 mM Na citrate pH 5.6. Following cryo-protection in 2.5 M Na malonate, these crystals diffracted to 3.0 Å resolution (Table 1) at Diamond Light Source (Didcot, UK) beamline I04 and contained two copies of the complex in the asymmetric unit. Data was reduced using iMOSFLM (28) followed by SCALA (29). An atomic model was generated by molecular replacement using Sac3^757–787^:Sus1B from PDB 3FWB (17) as a search model using PHASER (29) and Nup1 residues 326–340 built into the *Fo-Fc* difference density. The final model was obtained after iterative cycles of refinement, using PHENIX (30) and rebuilding with COOT (31). Crystals with *P2_1_* symmetry were obtained when Nup1^316–340^ was mixed in a 7:1 molar ratio with the Sac3^753–805^:Sus1B:Cdc31 complex (14.6 mg/ml) in 16% w/v PEG600, 2% w/v PEG 1K, 10% v/v glycerol and 100 mM MES pH 6.0. Following cryo-protection in mother liquor supplemented with 20% w/v PEG400, these crystals diffracted to 2.6 Å resolution (Table 1) and contained two copies of the complex in the asymmetric unit. Data was reduced using XDS (32) and AIMLESS (29). A model was obtained by molecular replacement using PDB 3FWB as a search model and, although Nup1 residues 330–340 could be built unambiguously, electron density for residues 316–329 was absent. Both models had excellent geometry and had MolProbity (33) scores in the 100th percentile.

**Table 1.** Crystal data

*Crystal*	Sac3^757–787^:Sus1B:Nup1^322–355^	Sac3^753–805^:Sus1B:Cdc31:Nup1^316–340^
Symmetry	*P3_2_21*	*P2_1_*
Unit cell dimensions		
*a,b,c* (Å)	95.55, 95.55, 105.66	51.26, 62.38, 124.51
α, β, γ (°)	90, 90, 120	90, 98.53, 90
**Data collection**		
Wavelength (Å)	0.9795	0.9795
Resolution range (Å)^a^	48–3.0 (3.16–3.0)	43.8–2.61 (2.74–2.61)
Total observations^a^	89,106 (13269)	116,371 (14,559)
Unique observations^a^	11,573 (1667)	23,841 (2,896)
Completeness (%)^a^	100 (100)	99.8 (99.9)
Multiplicity^a^	7.7 (8.0)	4.9 (5.0)
*R*_pim_^a^	0.05 (0.38)	0.038 (0.361)
Mean I/σ(I)^a^	11.8 (2.4)	13.2 (2.5)
**Refinement**		
*R*_cryst_/*R*_free_ (%)	20.7/24.4	20.0/23.6
Bond length r.m.s.d. (Å)	0.004	0.004
Bond angle r.m.s.d. (°)	0.66	0.83
MolProbity score / percentile	0.96/100	1.3/100
Ramachandran plot (%)		
Favoured	99.3	99.5
Allowed	0.7	0.5
Forbidden	0	0

^a^Parentheses refer to final resolution shell.

### Construction of yeast strains

Supplementary Table SI lists the yeast strains used in this study. All strains constructed were based on strain YGC242 (transformed with the pASZ11-NupNop plasmid; *ADE2*) (34) into which a *SAC3*-containing DNA product encompassing the *URA3* marker was introduced by homologous recombination. The DNA product was constructed by overlap extension PCR using Herculase II Fusion DNA polymerase (Agilent Technologies) that contained the following features (5’ to 3’): *SAC3* promoter region (from 422 bp upstream of start ATG) and *SAC3* full-length coding sequence (S288C chromosome IV; 771,455–775,779), Gly4 linker, mTurquoise2 coding sequence, STOP codon, *ADH1* terminator (S288C chromosome XV; 159,328–159,547), *URA3* marker, *SAC3-SSY1* intergenic region and *SSY1* coding sequence up to bp 383 (S288C chromosome IV; 775,783–776,544). For PCR, *Saccharomyces cerevisiae* genomic DNA (Novagen; Beston, UK) was used as template when amplifying chromosomal sequences (as indicated above). Plasmid pRS416 (35) was used as template for amplification of the *URA3* marker (nucleotides 182–1298, which contained *S. cerevisiae URA3* promoter, coding sequence and terminator), whereas pmTurquoise2-H2A (plasmid 36207, Addgene) was used for amplifying the mTurquoise2 coding sequence (nucleotides 4390–7). The fully constructed 7157 bp PCR product was cloned into blunt-end ligation vector pCRBlunt (Invitrogen) and the entire insert verified by sequencing. Mutations were introduced to this plasmid using Quikchange mutagenesis (Invitrogen) according to a modified protocol (36). A linear fragment for homologous recombination was released by digestion with the unique restriction endonucleases SnaBI and SacI which cut at bp 93 and bp 7142, respectively, within the insert. Following homologous recombination with yeast strain YGC242 (34) using standard methods (37), selection was performed on SD–Adenine–Uracil plates and genomic DNA extracted from a number of putative clones by using Wizard Genomic DNA purification kit (Promega). Genomic DNA was subjected to PCR (using a forward primer that had the same sequence as nucleotides 770,629–770,651 of chromosome IV and a reverse primer spanning the mTurquoise2-STOP-*ADH1* terminator region) to confirm correct integration. The purified PCR product was sequenced to verify the integrity of key features and the presence of expected mutations. Quantitative PCR (Supplementary Table S2) confirmed that the levels of *SAC3* transcript in the three mutated strains (DJ44 L768A-H774A, DJ45 L768A-H774D and DJ46 F772A-H774A) were comparable to those in the wild-type strain (DJ43).

### Yeast growth media

Selection of *URA3*^+^ strains was performed on SD–Adenine–Uracil plates. Following verification of strains (see above), cells were grown in SD–Adenine (to maintain selection of the pASZ11:NupNop plasmid) for all experiments. SD–Adenine (pH 5.8) was formulated as follows (quantities of components per L are indicated): Yeast nitrogen base without amino acids (Formedium, Hunstanton, UK), 6.7 g; D(+) glucose, D(+) raffinose or D(+) galactose (Formedium, Hunstanton, UK), 20 g; Bacto-agar (for plates; Becton Dickinson and Co., MD, USA), 20 g; L-Isoleucine, 30 mg; L-Valine, 150 mg; L-Arginine-HCl, 20 mg; L-Histidine-HCl monohydrate, 20 mg; L-Leucine, 100 mg; L-Lysine-HCl, 30 mg; L-Methionine, 20 mg; L-Phenylalanine, 50 mg; L-Threonine, 200 mg; L-Tryptophan, 20 mg; L-Tyrosine, 30 mg; L-Glutamic acid, 20 mg; L-Aspartic acid, 20 mg; L-Serine, 100 mg and Uracil, 20 mg.

### Yeast growth assays

Cells were grown overnight in SD–Adenine medium containing 2% raffinose, diluted in water to OD_600_ of 0.1 and then further diluted 10-fold serially and spotted on to SD–Adenine plates containing 2% glucose. Plates were incubated at the indicated temperatures and digitized as indicated.

### Fluorescence microscopy

For *in vivo* Sac3 localisation, cells were grown in SD–Adenine (2% glucose) to mid log phase and then resuspended in PBS supplemented with 2% glucose. Microscopy was performed on an Andor Revolution XDi spinning disk confocal system built around an inverted Nikon Eclipse Ti microscope with a 100× 1.4 NA oil immersion objective and running under iQ 2.7 software (Andor). mTurquoise2 was excited at 445 nm and EGFP at 488 nm with appropriate filter sets used for each. Use of 445 nm laser light for mTurquoise2 reduced cross-excitation of EGFP considerably and, together with ECFP-specific filter sets, eliminated GFP (green fluorescent protein) bleed-through into the CFP (cyan fluorescent protein) channel allowing near complete discrimination of signals originating from Nup49 and Sac3.

For fluorescence *in situ* hybridisation, cells were grown overnight at 30°C in SD–Adenine (2% glucose), then diluted into fresh medium, grown to a OD_600_ of ∼0.4, temperature shifted to 37°C (if appropriate) and grown for a further 3 h to an OD_600_ of ∼1.0 before being processed. Cell cultures were fixed with 4% formaldehyde (at the growth temperature) and then in 0.1 M potassium phosphate buffer pH 6.5 at room temperature and processed further as described (18). Images were obtained using a 63× 1.4 NA oil immersion objective on a Zeiss LSM 780 inverted confocal microscope running under ZEN 2010 software (Carl Zeiss Ltd). DAPI (4',6-diamidino-2-phenylindole) was excited at 405 nm and Cy3 at 514 nm with appropriate detection settings. Two hundred cells were examined for each mutant and temperature; similar results were obtained in a second series.

The *GAL7-GAL10-GAL1* gene cluster was imaged using cells grown overnight in SD–Adenine containing either 2% glucose (for repression) or 2% raffinose (for de-repression or activation experiments), diluted to an OD_600_ of 0.1 in 2% glucose (repression), galactose (activation) or raffinose (de-repression) containing medium and grown for a further 4 h. Cells were resuspended in PBS supplemented with 2% of the appropriate sugar and spread onto microscope slides coated with a media patch containing 2% agarose and 2% of the same sugar. Microscopy was performed with a Zeiss LSM780 inverted confocal microscope and was limited to 1 h after mounting. EGFP was excited at 488 nm and Z stacks (five slices) acquired to step through the majority of the nuclear volume of the cells in the field of view. Numerous stacks were acquired so that the total number of cells analysed was ∼100 per replicate. For each cell, the slice in which the *GAL* dot was brightest was selected for localisation and the *GAL* dot was scored as peripheral if there was overlap between its centre and the Nup49 signal as described (38). All fluorescence images were processed with ImageJ 1.46r (NIH).

## RESULTS

### The TREX2 CID domain complex binds between Nup1 residues 322 and 370

Previous work indicated that Nup1, Nup60 and the Sac3^CID^ domain complex (Sac3^723–805^ bound to Sus1A, Sus1B and Cdc31) contribute to the localisation of TREX2 to NPCs (12,14,16–17). Pull-down assays using a series of Nup1 fragments indicated that the Sac3^CID^ domain complex bound to Nup1 residues 322–370 (Supplementary Figure S1A) but did not bind to Nup60 (Supplementary Figure S2). The structure of the Sac3^CID^ domain complex (17) was then used to engineer a series of CID domain sub-complexes based on Sac3 fragments that contained binding sites for one or two of the Sus1A, Sus1B or Cdc31 partner chains (Supplementary Figure S1B) and their binding to Nup1^191–385^ assessed using pull-down assays (Supplementary Figure S1C). Nup1^191–385^ bound the complete Sac3^CID^ complex and all sub-complexes that contained Sus1B, whereas it did not bind complexes that contained only Sus1A or Cdc31. Overall, these data indicated that the Sus1B chain and the region of Sac3 bound to it (residues 757–787) contained the major determinants for Nup1 binding.

### Crystal structures of the Nup1-containing complexes

A series of fragments based on Nup1^322–370^ were used in crystallisation trials. Because the binding of these Nup1 fragments to Sac3^757–787^:Sus1B or Sac3^753–805^:Sus1B:Cdc31 was not sufficiently strong to enable stoichiometric complexes to be isolated by size exclusion chromatography, the Nup1 fragments were purified separately and added in a ∼6-fold molar excess. Crystals with *P3_2_21* symmetry that diffracted to 3.0 Å resolution were obtained from a solution containing Nup1^322–355^ and Sac3^757–787^:Sus1B and crystals with *P2_1_* symmetry that diffracted to 2.6Å resolution were obtained with Nup1^316–340^ and the Sac3^753–805^:Sus1B:Cdc31 complex (Table 1). Molecular replacement using appropriate search models derived from the Sac3^CID^ domain complex (17) showed clear difference density due to the Nup1 fragment, with the side chains of Phe336 and Phe338 being especially clear. For the *P2_1_* crystals, Nup1 residues 330–339 were easily built, but density for residues 316–329 was not observed, even after repeated cycles of refinement and rebuilding. After refinement, additional weak density was observed between the N- and C-terminal helices of each Sus1 chain that appeared to be due to a second weakly bound peptide chain that was tentatively assigned to Nup1 residues 326–330. For the *P3_2_21* crystals, Nup1 residues 326–340 were easily built into the difference density, albeit the Nup1 chains crossed from one Sac3:Sus1 complex in the asymmetric unit to the other in a form of domain swapping (39) (Supplementary Figure S3C). Overall, the density of Nup1 residues 334–340 was much clearer than the remainder (Supplementary Figure S3A and B), consistent with residues 326–333 being bound more weakly. After iterative cycles of refinement and rebuilding, a final *2Fo-Fc* map with excellent clarity was obtained (Supplementary Figure S3D). Density that could be assigned to residues 341–355 was absent from the final *2Fo-Fc* and *Fo-Fc* maps. Vague density was visible in the place assigned to Nup1 residues 326–331 in the *P2_1_* crystal and it appears that in both crystals an additional Nup1 peptide chain became bound at this site. In both cases, the density due to this additional peptide chain was poor, consistent with it being bound weakly and/or sub-stoichiometrically and is likely the result of the peptide being present in molar excess during crystallogenesis, indicating that the binding of this second Nup1 chain was likely a crystallisation artefact. In the final model of both complexes, the structural arrangement of Sus1B bound to Sac3 and Cdc31 bound to Sac3 was essentially unaltered from that observed in the absence of Nup1 (17), consistent with Nup1 binding not generating a substantial conformational change.

**Figure 1.**
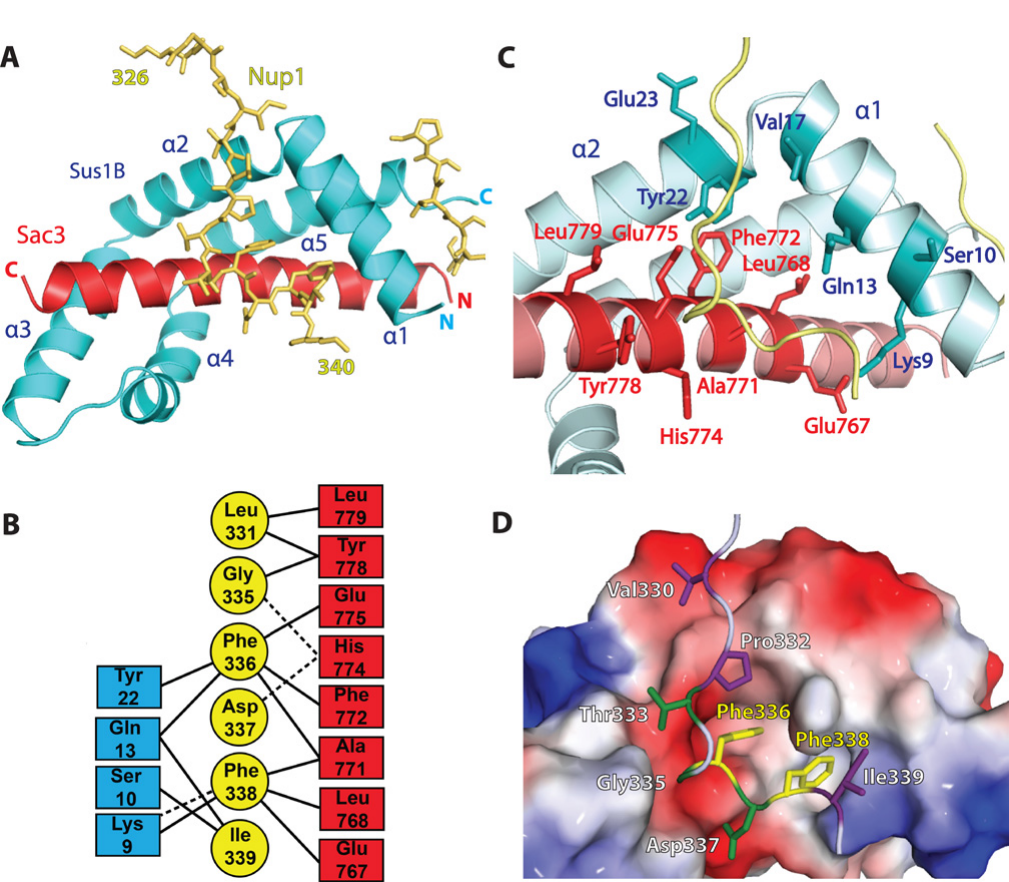
The structure of the Nup1:Sac3^757–787^:Sus1B complex. (**A**) Overview of the arrangement of the chains in the complex. (**B**) Schematic representation of the interactions formed between Nup1, Sac3 and Sus1B upon complex formation. Dashed lines represent putative hydrogen bonds or salt bridges. (**C**) Principal interacting residues in Sac3 (highlighted in red) and Sus1B (highlighted in dark cyan) that are buried in the interface with Nup1. (**D**) Electrostatic surface representation of Sac3 and Sus1B in the same view as in (C) showing the cavity to which Nup1 binds. Nup1 residues interacting with only Sus1 are shown in purple; those interacting with only Sac3 in green and those interacting with both are shown in yellow.

In both crystals, the principal Nup1 binding site was based on a cavity formed by Sus1B helices α1 and α2 and residues 757–787 of the Sac3^CID^ domain helix (Figure 1A,C,D). The Nup1 chain adopted an unstructured conformation (Figure 1A) and the interaction was dominated by burying the aromatic side chains of Phe336^Nup1^ and Phe338^Nup1^. A substantial number of residues on Sus1B and Sac3 were buried in the interaction interface (Figure 1B and C) with Nup1 burying ∼410 Å^2^ and ∼330 Å^2^ of surface area with Sus1 and Sac3, respectively. The binding cavity had a mixed charge character, with Lys9^Sus1^ making the opening of the cavity nearest to the N-terminus of Sus1 helix α1 positively charged and Glu775^Sac3^ making the opposite face of the cavity negatively charged (Figure 1C and D). The interior of the binding cavity contained two deeper hydrophobic clefts into which the aromatic side chains of Phe336^Nup1^ and Phe338^Nup1^ fitted snugly (Figure 1D). The cavity that accommodated Phe336^Nup1^ was formed from the juxtaposition of side chains from Sus1 Gln13, Leu16 and Tyr22 and Sac3 Leu768, Ala771 and Phe772, whereas the cavity that accommodated Phe338^Nup1^ was formed by the juxtaposition of side chains from Sus1 Lys9, Ile12 and Gln13 and Sac3 Met764, Glu767 and Leu768 (Figure 1C and D). Many of the cavity-forming residues in Sus1 and Sac3 made close contacts with the two Nup1 phenylalanines (Figure 1B) and the importance of these two hydrophobic aromatic residues in complex formation was confirmed by engineering a Nup1 mutant in which these two residues were mutated to Ala that eliminated binding to the Sac3^CID^ domain complex (Supplementary Figure S4).7

Although Phe336^Nup1^ and Phe338^Nup1^ were almost entirely buried in the interaction interface, anchoring Nup1 to the Sac3^CID^ complex, the interaction was augmented through additional contacts, especially by His774^Sac3^ that formed a putative salt bridge with Asp337^Nup1^ and a hydrogen bond with Gly335^Nup1^ (Figure 1B–D). Additionally, Tyr22 at the N-terminal end of helix α2 in Sus1 was stacked against Pro332^Nup1^ as the Nup1 chain descends into the cavity (Figure 1C and D). The ends of the Nup1 chain that bind to the cavity were held in place through contacts between Val330^Nup1^ with Glu23^Sus1^ and Ile339^Nup1^ with Lys9^Sus1^ and Ser10^Sus1^ (Figure 1B–D). The overall arrangement of helices in the Sac3:Sus1B region with and without Nup1 bound was similar (backbone Cα r.m.s.d. of 1.28 Å), albeit Lys9, Gln13 and Tyr22 in Sus1 and Phe772^Sac3^ adopted different rotamer conformations to accommodate Nup1 Phe336 and Phe338 (Supplementary Figure S5A-C). Despite the structural similarity of Sac3^723–756^:Sus1A and Sac3^757–787^:Sus1B (17), only Sac3^CID^ complexes containing Sus1B bound Nup1 (Supplementary Figure S1C). This appeared to be due to the Sac3:Sus1A interface not generating a similar binding cavity and derived primarily from differences in the Sac3 helix (Supplementary Figure S5D and E). Thus, Ala771^Sac3^ and Leu768^Sac3^ in the Sac3:Sus1B interface are replaced by the more bulky Asp733^Sac3^ and Ile730^Sac3^, respectively, in the Sac3:Sus1A interface. These more bulky side chains, together with the presence of Leu734^Sac3^ results in the cavity formed at the Sac3:Sus1A interface being much flatter than that formed by Sac3:Sus1B and lacking the hydrophobic clefts into which Phe336^Nup1^ and Phe338^Nup1^ bind (Supplementary Figure S5D and E).

**Figure 2.**
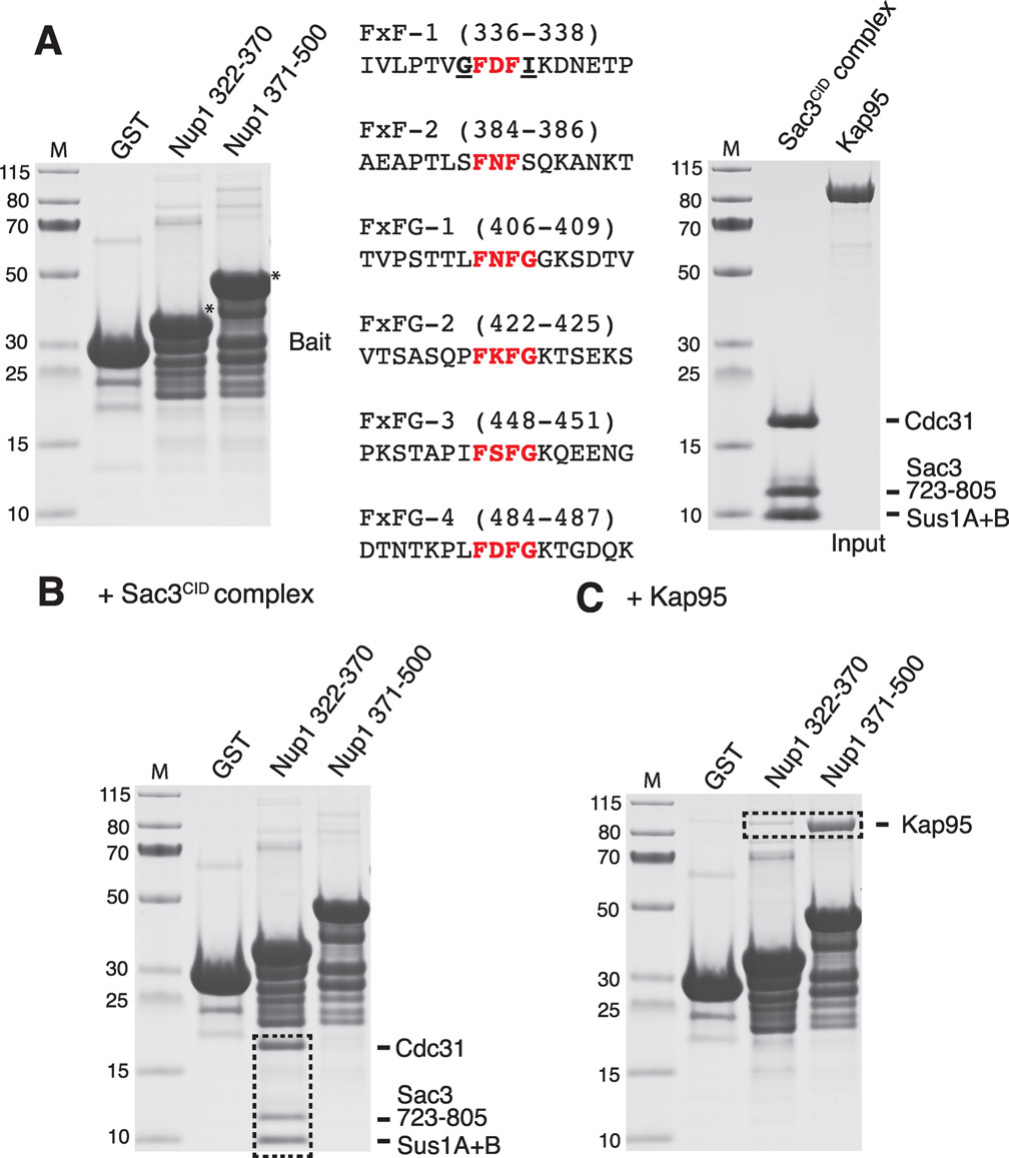
The binding of karyopherin Kap95 and the Sac3^CID^ domain complex to FxF and FxFG motifs within Nup1. (**A**) GST, GST-Nup1^322–370^ and GST-Nup1^371–500^ were immobilised on Glutathione Sepharose resin as bait for pull-down assays with purified Kap95 and Sac3^CID^ domain complex used as input. Full-length GST-Nup1 material is marked by an asterisk with the sequence of FxF and FxFG motifs present shown (motif core in red and numbering starting with the first Phe). (**B, C**) Bound material remaining after incubation, resin washing, SDS-PAGE and Coomassie staining is shown boxed. Whereas the Sac3^CID^ domain complex bound only the FDFI motif, Kap95 bound only the FxFG motifs. M, molecular weight markers (kDa).

The arrangement of the side chains of Nup1 Phe336 and Phe338 was strikingly similar to that in which FxFG chains are arranged when bound to several nuclear transport factors (25,40–42). Pull-down assays (Figure 2 and Supplementary Figure S1A) showed that whereas the Sac3^CID^ complex bound to Nup1^322–370^ (that contains the FDFI motif that binds TREX2; FxF-1), it did not bind to Nup1^371–500^ (that contains another FxF motif and four FxFG repeats; Figure 2A and B). Conversely, the protein import karyopherin Kap95 bound to Nup1^371–500^ but interacted only very weakly with Nup1^322–370^ (Figure 2C). Furthermore, the Sac3^CID^ complex failed to bind to the numerous FxFG repeats in channel nucleoporin Nsp1 (Supplementary Figure S6). Together, these data indicate that the two Phes in Nup1^322–370^ are a specialised site for anchoring TREX2 to NPCs rather than serving as a binding site for transport factors.

### Structure-guided Sac3 mutations that impair Nup1 binding *in vitro* without compromising Sac3^CID^ complex assembly

**Figure 3.**
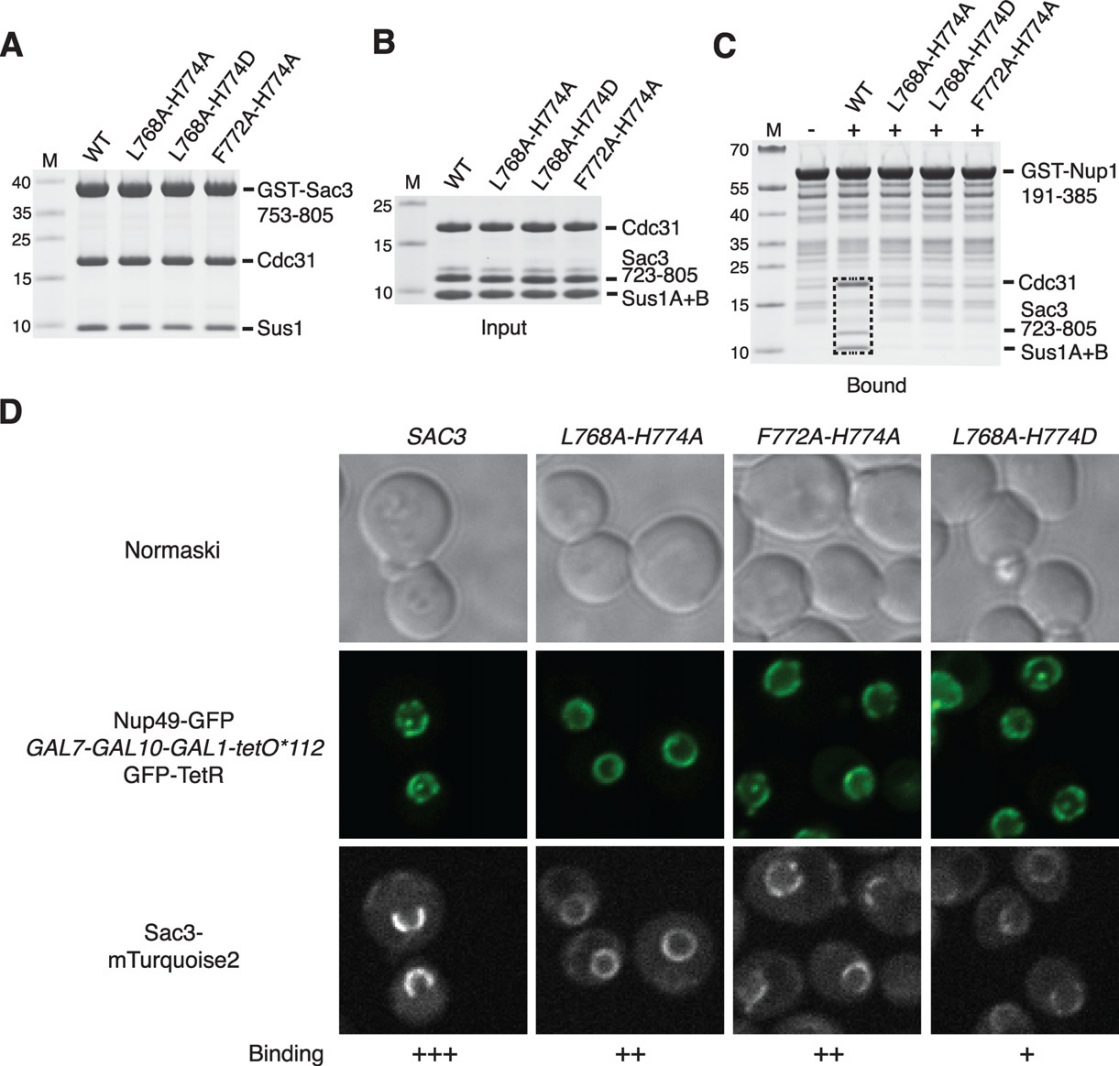
Mutations in Sac3 that impair binding of the Sac3^CID^ domain complex to Nup1 *in vitro* while not impacting on Sac3^CID^ complex assembly impair Sac3 targeting to the nuclear periphery *in vivo*. (**A**) Assembly of mutant Sac3^CID^ domain complexes were monitored by pull-down assays. Clarified bacterial lysates containing GST-Sac3^753–805^ wild-type (WT) or mutant derivatives co-expressed with Cdc31 were mixed with an excess of clarified bacterial lysate containing Sus1 and incubated with Glutathione Sepharose resin. Washed resins were analysed by SDS-PAGE and Coomassie staining. (**B**) Mutants were purified in a fragment of Sac3 encompassing residues 723–805 complexed to Sus1A, Sus1B and Cdc31 (the complete Sac3^CID^ domain complex) and equimolar amounts of each were used as input in pull-down assays using GST-Nup1^191–385^ as bait immobilised on Glutathione Sepharose resin (**C**). Washed resins were analysed by SDS-PAGE and Coomassie staining and demonstrated that Nup1 bound only to WT complex (boxed). M; molecular weight markers (kDa). (**D**) Fluorescence micrographs of yeast strains in which the sole genomic copy of *SAC3* carried a C-terminal mTurquoise2 tag and was either WT or mutated as indicated. The nuclear periphery was visualiszed by Nup49-GFP. NPC binding was most strongly impaired by *sac3 L768A-H774D*.

The structure of the interface between Nup1 and the Sac3^CID^ complex was used to engineer mutations in Sac3 that impaired Nup1 binding without impacting on the assembly of the Sac3^CID^ complex itself (so that Cdc31 and Sus1 remained bound). Leu768^Sac3^ and Phe772^Sac3^, which are central in forming the clefts into which Phe336^Nup1^ and Phe338^Nup1^ become buried and which form critical contacts with the Nup1 Phes, were mutated to Ala and His774^Sac3^ was mutated to either Ala (to prevent salt bridge formation to Asp337^Nup1^) or Asp (to introduce repulsion) (Figure 1B–D). A range of engineered mutants were expressed in the context of Sac3^753–805^ and tested for their ability to bind to Sus1B and Cdc31 (Figure 3A). These Sac3 variants all bound to both Sus1B and Cdc31 to levels comparable to wild-type Sac3, indicating that these mutations had not impaired Sac3^CID^ complex assembly. Furthermore, when these mutations were introduced into Sac3^723–805^, the complexes formed with Sus1A, Sus1B and Cdc31 were indistinguishable by SDS-PAGE from those formed by wild-type Sac3 (Figure 3B). However, Sac3^CID^ complexes containing these engineered mutations displayed considerably reduced Nup1 binding *in vitro* compared to the wild-type complex (Figure 3C).

### Sac3^L768A-H774D^ impaired TREX2 localisation to NPCs *in vivo* and reduced the nuclear periphery association of both de-repressed and activated GAL1

**Figure 4.**
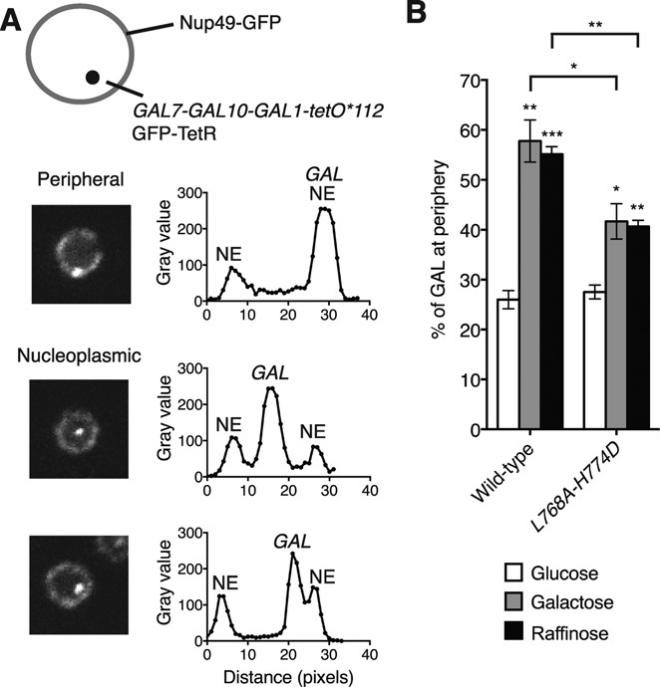
Inhibition of the Nup1:TREX2 interaction produces a defect in the relocation of the *GAL* gene cluster to the nuclear periphery under both de-repressed and activating conditions. (**A**) Schematic representation of a nucleus in which the *GAL* locus is labelled with a *tetO* array allowing visualisation with GFP-TetR and the nuclear periphery is marked by Nup49-GFP. Fluorescence micrographs of nuclei in which the *GAL* locus is positioned at the periphery or within the nucleoplasm and the corresponding plots of pixel intensity (gray value) versus distance (pixels). NE, nuclear envelope. (**B**) Percentage of cells in which the *GAL* locus is observed at the nuclear periphery with glucose, galactose and raffinose carbon sources for cells containing wild-type *SAC3* and the *L768A-H774D* variant. Error bars represent standard error of the mean for three independent experiments in which at least 100 cells were scored in each. An unpaired, two-tailed Student's *t* test was used to determine statistical significance; *P < 0.05, **P < 0.01, ***P < 0.001. *P* values represent a comparison between % of cells in which the *GAL* locus is observed at the nuclear periphery in glucose with other carbon sources for wild-type and mutant backgrounds, or as indicated.

NPC-bound TREX2 and gene bound-SAGA are thought to co-operate to promote the re-localisation of a subset of active genes, including the *GAL7-GAL10-GAL1* cluster, to the nuclear periphery upon activation with maintenance of an NPC-proximal position conferring optimal transcriptional activity (3,7–8). To test the functional significance of TREX2 binding to Nup1 *in vivo*, haploid yeast strains were constructed based on the strain YGC242 employed previously to construct high-resolution statistical maps of the *GAL7-GAL10-GAL1* gene cluster (34). YGC242 contains 112 *tetO* repeats inserted in the intergenic region between *GAL1* and *FUR4* and expressed a GFP-TetR fusion, allowing visualisation of the *GAL* locus (Figure 4A), whereas to visualise the nuclear periphery, the genomic copy of NPC component *NUP49* has been deleted and replaced by a GFP fusion expressed from plasmid pASZ11. Homologous recombination was used to introduce mTurquoise2 (ECFP variant (43)) at the C-terminus of the genomic copy of *SAC3* in this strain (to facilitate visualisation independently of Nup49) and also to introduce mutations into *SAC3* as appropriate.

Cells carrying wild-type *SAC3* showed a strong punctate nuclear periphery localisation of Sac3 (Figure 3D), often having a horseshoe-like shape similar to that seen with Mlp1 which is excluded from areas close to the nucleolus (44). *SAC3* variants showed a graded nuclear envelope staining defect: both the *L768A-H774A* and *F772A-H774A* variants showed reduced Sac3 peripheral localisation whereas *sac3 L768A-H774D* showed a greater reduction, with only a small amount of Sac3 observed at the nuclear periphery.

The *sac3 L768A-H774D* variant was then used to investigate the influence of impairing the binding of TREX2 to NPCs on the localisation of de-repressed or activated *GAL1* to the nuclear periphery. Consistent with previous work (7,22,34,38), we found that in the presence of glucose (repressed), cells containing wild-type *SAC3* had the *GAL* locus located at the periphery in 26 ± 2% of cells, which increased to 55 ± 1% of cells in the presence of raffinose (de-repressed) and to 58 ± 4% of cells in the presence of galactose (activated) (Figure 4B). For *sac3 L768A-H774D*, the proportion of cells showing the *GAL* cluster at the periphery in glucose (28 ± 2%) was similar to that for cells containing wild-type *SAC3*, whereas in the presence of either raffinose or galactose, the increase in cells that showed the *GAL* cluster at the nuclear periphery was substantially reduced to only 41 ± 1% (*P* < 0.01) and 42 ± 4% (*P* < 0.05), respectively (Figure 4B). These results demonstrate that the Nup1:TREX2 interaction is important for localising the *GAL* gene cluster to NPCs when it is either de-repressed or activated.

### Sac3^L768A-H774D^ generated cellular growth and nuclear mRNA export defects

**Figure 5.**
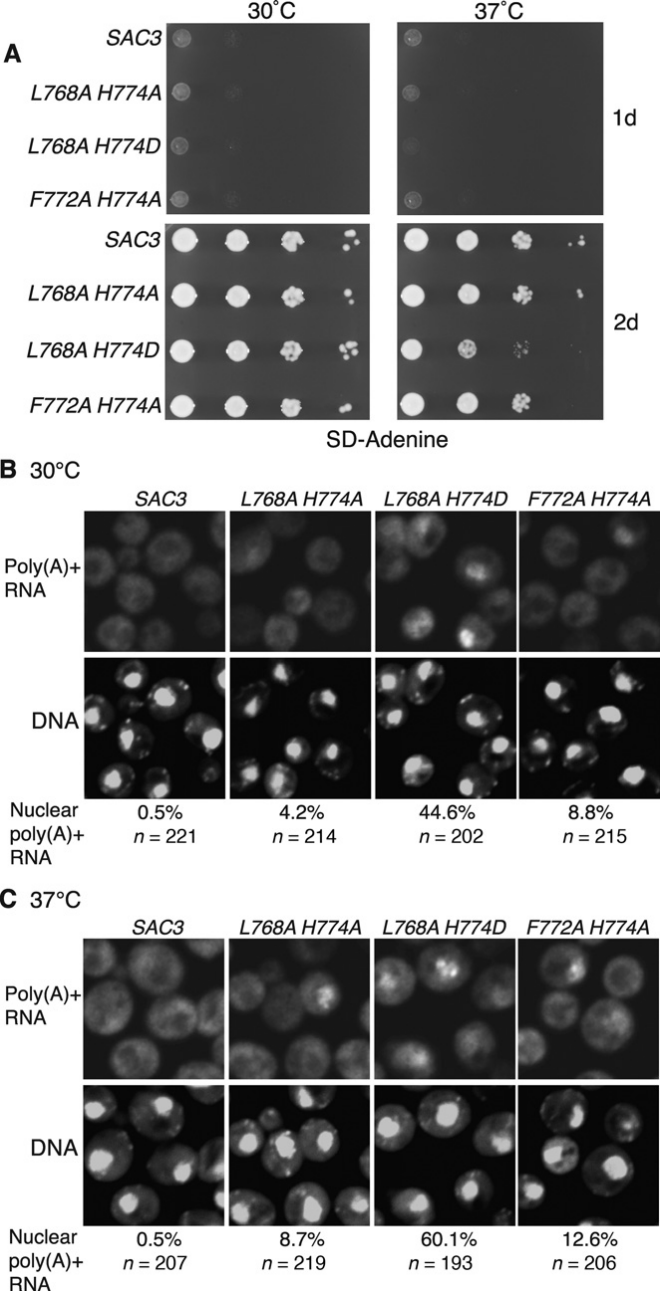
Inhibition of the Nup1:TREX2 interaction produces cellular growth and nuclear mRNA export defects. (**A**) Analysis of cellular growth by 10-fold serial dilution of indicated wild-type and *sac3* mutant strains on SD–Adenine (2% glucose). (**B,C**) Fluorescence *in situ* hybridisation analysis of poly(A)+ RNA export in the indicated wild-type and *sac3* mutant strains at 30 and 37°C. The number of cells analysed and the percentage showing nuclear poly(A)+ RNA is indicated. The *sac3 L768A-H774D* variant showed the most marked defects.

Impairing the Nup1:TREX2 interaction also generated defects in cellular growth and nuclear mRNA export. Although growth of all of the *sac3* variants was indistinguishable from wild-type at 30°C, at 37°C *sac3 L768A-H774D* showed reduced growth compared to wild-type (Figure 5A). Furthermore, the *sac3* variants showed differing degrees of nuclear mRNA accumulation (Figure 5B and C). At 30°C, although the *L768A-H774A* and *F772A-H774A* variants showed only slight nuclear accumulation of poly(A)+ RNA, *sac3 L768A-H774D*, in which NPC binding was most impaired, generated a greater degree of nuclear mRNA accumulation (Figure 5B). At 37°C, the proportion of cells displaying nuclear poly(A)+ accumulation increased for all the variants, with over 60% of cells that carried the *L768A-H774D* mutation showing an mRNA export defect (Figure 5C). Overall, the extent of poly(A)+ RNA accumulation in the nucleus correlated with the magnitude of growth defects observed (Figure 5A) and to the extent TREX2 was mislocalized from NPCs (Figure 3D).

## DISCUSSION

The crystal structures of Nup1 fragments bound to either Sac3^753–805^:Sus1B:Cdc31 or Sac3^757–787^:Sus1B (Figure 1) identified the molecular details of the interaction interface and enabled a *sac3* variant to be engineered that impaired the binding of TREX2 to NPCs while retaining the structural integrity of the complex (Figure 3). This *sac3* variant enabled the function of the NPC:TREX2 interaction to be probed selectively *in vivo* and has established its importance in the localisation of both de-repressed and activated *GAL1* to NPCs (Figure 4) and also in bulk mRNA nuclear export (Figure 5). Previous studies employed deletions in the long Sac3^CID^ helix encompassing the Sus1A, Sus1B and Cdc31 binding sites (17) or deleted the whole CID domain or C-terminal region (14,16). In each case, mRNA export was impaired to varying degrees, depending on the severity of the mutation employed and, with deletions of the whole CID domain and C-terminal region of Sac3, TREX2 was mis-localised and no longer present primarily at NPCs. However, it was unclear whether the accumulation of poly(A)+ RNA in these mutants resulted from loss of NPC association of the TREX2 complexes, or because of the absence of one of Sac3's partner chains (such as Sus1B), or because deletion of large regions of Sac3 resulted in a non-functional TREX2 complex. Studies that employed *nup1Δ* strains (7,22) were also difficult to interpret because the pleiotropic nature of the defects introduced by deletion of *NUP1* (23) made it difficult to differentiate between phenotypes generated by the mis-localization of TREX2 from those resulting from defects introduced into other nuclear transport pathways. Our present study circumvented these problems by engineering structure-guided mutations in Sac3 that impaired only its NPC association but left the TREX2 complex intact and did not interfere with other Nup1 functions. With our *sac3* variants, because neither assembly of the TREX2 complex nor the other functions of Nup1 were compromised, our results indicate that NPC binding of TREX2 is a requirement for efficient export of bulk nuclear mRNA.

The observation that *sac3 L768A-H774D* still showed some residual NPC association (Figure 3D) would be consistent with additional regions outside of the Sac3^CID^ domain contributing to NPC binding (14). Nevertheless, the Nup1 region identified here makes a major contribution to binding and introduction of mutations in the Sac3^CID^ helix that interfered with binding to this region of Nup1 was sufficient to impair the association of TREX2 with NPCs *in vivo.* The level of impairment generated was sufficient to produce defects in NPC *GAL1* localization, growth and mRNA export (even though impairing mRNA export often does not impact on cellular growth (45)) and thus our results establish the functional importance of the TREX2:NPC interaction in *S. cerevisiae*.

**Figure 6.**
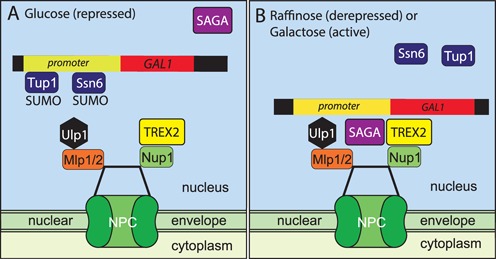
Schematic illustration of the function of TREX2 binding to Nup1 in regulating *GAL1*. (**A**) When glucose is available as a carbon source, Mig1 results in *GAL1* being repressed together with promoter-bound Ssn6 and Tup1, both of which are sumoylated. (**B**) Under either de-repressing (raffinose) or activating (galactose) conditions, the SUMO protease Ulp1, that is bound to NPCs, removes the SUMO moieties from Ssn6 and Tup1 relieving repression (19). The action of Nup1-bound TREX2 and chromatin-bound SAGA in retaining *GAL1* at the NPC ensures that repression by Ssn6 and Tup1 is suppressed because of proximity to Ulp1. The proximity of *GAL1* and other highly regulated actively transcribing genes to the NPC may facilitate the nuclear export of transcripts as well as facilitating transcriptional memory and promoting gene stability (1,3,4,7,8,9,10,11).

The results obtained with *sac3 L768A-H774D* in which TREX2:NPC association was impaired indicated that TREX2 facilitated the NPC association of the *GAL* gene cluster, both when repression was removed (in raffinose), and also when the gene was activated in the presence of galactose. The relocation of *GAL1* to the nuclear periphery following either de-repression or activation (Figure 6) is thought to make a key contribution to its regulation and also to gene stability and transcriptional memory. Analogous mechanisms are also probably employed for other highly regulated, actively transcribing genes such as *INO1* and *HXK1* (1,3–4,7–11). Glucose-induced repression is mediated by Mig1 in conjunction with sumoylated gene-bound co-repressors Ssn6 and Tup1. De-repression is mediated by NPC-bound SUMO protease Ulp1 that desumoylates Ssn6 and Tup1, releasing them from the promoter and initiating chromatin remodelling and binding of SAGA (19). Thus under de-repressive or inducing conditions, when glucose is replaced by another carbon source, localising the *GAL* cluster to NPCs facilitates de-repression and subsequent transcription by desumoylating chromatin-bound gene regulators ((19) and Figure 6B). Under repressive conditions, inducible genes are thought to be located away from NPCs and so inaccessible to Ulp1, allowing sumoylation of regulators to be maintained ((19) and Figure 6A). Although TREX2 could facilitate de-repression by mediating the movement of the *GAL* locus to NPCs, it is perhaps more likely that TREX2 instead functions primarily to retain de-repressed genes at NPCs to prevent them becoming repressed by binding of sumoylated Ssn6 and Tup1 generated elsewhere in the nucleus (Figure 6). Further work will be required to dissect these possibilities. Overall, our results are consistent with the proposal that NPCs have a dual function in promoting transcription, facilitating relief of repression and promoting activation, thereby providing spatial and temporal clues for optimal regulation of inducible genes (19).

The observation that Phe336^Nup1^ and Phe338^Nup1^ made such a large contribution to the primary TREX2:Nup1 interaction interface (Figure 1 and Supplementary Figure S4) was reminiscent of the way in which nucleoporin FxFG repeats bind to nuclear transport factors (25,40–42). However, there was a critical difference between the transport factor interactions and that of TREX2 in that the TREX2-binding motif (FDFI in Nup1^322–370^; Figure 2A, FxF-1) had an Ile in place of the Gly in the FxFG motif that is necessary to enable the nucleoporin chain to turn sharply to accommodate the shape of FG binding pockets in most transport factors. Indeed, Kap95 showed little affinity for Nup1^322–370^ whereas TREX2 showed little affinity for nucleoporin FxFG repeats in Nup1 (Figure 2 and Supplementary Figure S1A) or Nsp1 (Supplementary Figure S6). The Nup1 FDFI motif that binds to TREX2 is the only motif to be immediately preceded by Gly (Figure 2A), allowing the Nup1 chain to turn abruptly into the binding pocket formed by Sac3 and Sus1B helices α1 and α2 and permitting the Phes to fit snugly into their binding clefts (Figure 1). None of the FxFG motifs tested in Nup1^371–500^ would allow the Nup1 chain to turn into the binding cavity in the same way as observed for the GFDFI motif. Therefore these structural features of the interaction interface account for TREX2 binding specifically to the FDFI motif but not to the FxFG motifs contained elsewhere in Nup1 or other nucleoporins, such as Nsp1. Overall, these data indicate that the two Phes in Nup1^322–370^ are a specialised site for anchoring TREX2 adjacent to the NPC transport channel and suggest that TREX2 is unlikely to act as a general transport factor because of its inability to interact with FG repeats within the channel itself.

Anchoring of TREX2 to the N-terminal region of Nup1 that located it in the nuclear basket close to the NPC transport channel may promote the export of mature mRNPs by docking and concentrating them near the transport channel in a manner analogous to docking of protein import and export complexes (46). Increasing the local concentration of transcripts in the vicinity of their exit site would increase the frequency with which they enter the channel and so promote export. Such a mechanism seems also to function in mammalian cells in which the Sac3 homologue, GANP (germinal centre-associated protein), has been proposed to chaperone mature transcripts and increase the efficiency of transport from transcription/processing factories deeper in the nucleus to NPCs (47,48). TREX2 may also have a more direct role in the maturation of partially processed transcripts, whereby the mRNA binding interface formed by TREX2 components Thp1 and Sem1 may facilitate loading of captured transcripts with Mex67:Mtr2 (which binds to the N-terminal region of Sac3 (14)) in concert with Yra1 and Sub2 (18,49) to facilitate generation of export-competent mRNPs at the NPC. Our data indicate that confining TREX2 to NPCs contributes to efficient mRNA export, suggesting that Mex67:Mtr2 loading of transcripts may become rate-limiting in the absence of the TREX2:NPC association.

The bulk poly(A)+ mRNA export defect observed when the Nup1:TREX2 interaction was impaired *in vivo* is not necessarily incompatible with data (22) that suggested that the NPC association of *GAL1* down-regulated transcription of the gene. Thus, when the association of active *GAL1* with NPCs was disrupted using either *ada2Δ* or *nup1Δ*, the levels of *GAL1* transcription increased (22). Furthermore, the kinetics of repression were significantly delayed following a shift to glucose in *ada2Δ* and *nup1Δ* strains, consistent with the gene-NPC tether facilitating rapid repression. However, Ada2 is a component of the SAGA complex and so, although this strain impaired the association of active *GAL1* with the nuclear envelope, it was not clear that it also impaired TREX2 binding to NPCs. Consequently, in the *ada2Δ* strain, TREX2 may still have been able to facilitate mRNA export independent of its localising active *GAL1* to NPCs. Unfortunately, because mRNA export is impaired in *nup1Δ* cells, it was not possible for these authors to assess the impact on this function of mislocalising TREX2 from NPCs. We circumvented this difficulty by employing Sac3 variants to impair the TREX2 and activated *GAL1* NPC association and so have avoided any influence of Nup1 on mRNA export.

In summary, the crystal structures of the Sac3^CID^ domain complex with Nup1 have defined the molecular basis of TREX2's primary interaction with the NPC and showed that Nup1 residues 330–340, and especially Phe336 and Phe338, bind to a cavity formed between Sac3 residues 757–787 and Sus1B helices α1 and α2. Structure-guided Sac3 variants that impair the Nup1:TREX2 interaction *in vitro* and *in vivo* were engineered and, in addition to impairing the localization of activated *GAL1* to NPCs, also exhibit both growth and bulk poly(A)+ mRNA nuclear export defects. Our results provide a structural context for understanding how the binding of TREX2 to NPCs is mediated and indicate that the interaction of TREX2 with NPCs is required for its function in orchestrating the integration of mRNA nuclear export with preceding steps in the gene expression pathway as well as the re-positioning of a subset of highly regulated and actively transcribing genes that facilitates control of their expression.

## ACCESSION NUMBERS

Coordinates and structure factors for the 3.0 Å resolution Sac3^757–787^:Sus1B:Nup1^322–355^ and the 2.6 Å resolution Sac3^753–805^:Sus1B:Cdc31:Nup1^316–340^ complexes have been deposited in the Protein Data Bank with accession numbers 4C31 and 4MBE, respectively.

## SUPPLEMENTARY DATA

Supplementary Data are available at NAR Online.

SUPPLEMENTARY DATA
