# Supplementary material for: Structural basis for binding the TREX2 complex to nuclear pores, GAL1 localisation and mRNA export
Source: Nucleic Acids Res. 2014 Apr 4;42(10):6686–97. doi: 10.1093/nar/gku252 (PMC4041426; doi:10.1093/nar/gku252)
Supplement: SUPPLEMENTARY DATA [file supp_42_10_6686__index.html]

Structural basis for binding the TREX2 complex to nuclear pores, GAL1 localisation and mRNA export — SUPPLEMENTARY DATA 

# Structural basis for binding the TREX2 complex to nuclear pores, *GAL1* localisation and mRNA export

## SUPPLEMENTARY DATA

**Files in this Data Supplement:**

- SUPPLEMENTARY DATA
