# Supplementary material for: Structural basis for binding the TREX2 complex to nuclear pores, GAL1 localisation and mRNA export
Source: Nucleic Acids Res. 2014 Apr 4;42(10):6686–97. doi: 10.1093/nar/gku252 (PMC4041426; doi:10.1093/nar/gku252)
Supplement: SUPPLEMENTARY DATA [file supp_gku252_nar-03599-m-2013-File008.pdf]

Structural basis for binding the TREX2 complex to nuclear pores, *GAL1* localization, and mRNA export

Divyang Jani, Eugene Valkov & Murray Stewart*

MRC Laboratory of Molecular Biology, Francis Crick Avenue, Cambridge Biomedical Campus, Cambridge CB2 0QH, UK.
*ms@mrc-lmb.cam.ac.uk

SUPPLEMENTARY INFORMATION

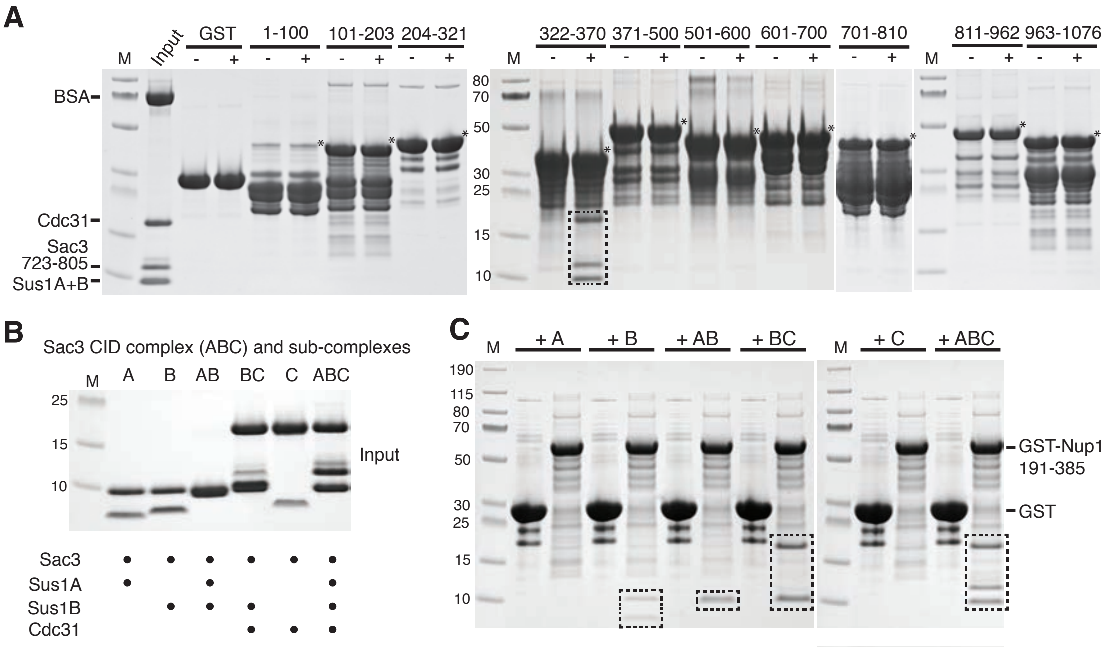

Supplementary Figure S1: Nup1 residues 322-370 interact directly with the Sac3^{CID} domain complex (Sac3$^{723\text{-}805}$ in complex with Cdc31, Sus1A and Sus1B) in which Sus1B and the region of Sac3 to which it is bound (residues 757-787) are major Nup1 binding determinants (A) The interaction between Nup1 and the Sac3^{CID} domain complex was probed by pull-down assays. GST and GST-Nup1 fragments spanning full-length Nup1 were immobilised on glutathione sepharose resin and were washed, after which purified Sac3$^{723\text{-}805}$:Cdc31:Sus1 complex in 0.5 % w/v BSA (input lane) was added and incubated. The resins were washed and analysed by SDS-PAGE and Coomassie staining. The GST-Nup1 fragments were highly susceptible to proteolysis but substantial quantities of full-length material (marked by an asterisk) could be obtained in each case, except for Nup1$^{1\text{-}100}$. Sac3^{CID} domain complex bound to Nup1$^{322\text{-}370}$ is shown boxed. **(B)** Purified Sac3^{CID} domain complex and sub-complexes, their corresponding abbreviated names and their composition. **(C)** Equimolar amounts of each complex were used as input for a pull-down assay in which GST and GST-Nup1$^{191\text{-}385}$ were immobilised on glutathione sepharose resin. The various purified Sac3^{CID} domain sub-complexes were added, incubated and the resins washed and analysed by SDS-PAGE and Coomassie staining. The Sac3^{CID} domain complexes that bound to Nup1$^{191\text{-}385}$ are boxed. M; molecular weight markers (kDa).

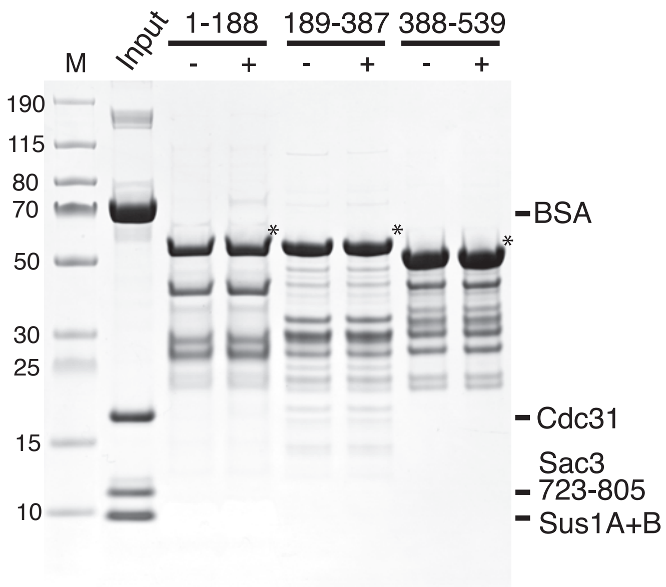

Supplementary Figure S2: Nup60 does not interact directly with the Sac3^{CID} domain complex GST-Nup60 fragments spanning full-length Nup60 were immobilised on glutathione sepharose resin and washed, after which purified Sac3$^{723-805}$:Cdc31:Sus1 complex in 0.5 % w/v BSA (input lane) was added and incubated. The resins were washed and resin-bound material analysed by SDS-PAGE and Coomassie staining. The GST-Nup60 fragments were susceptible to proteolysis but substantial quantities of full-length material (marked by an asterisk) could be obtained in each case. M; molecular weight markers (kDa).

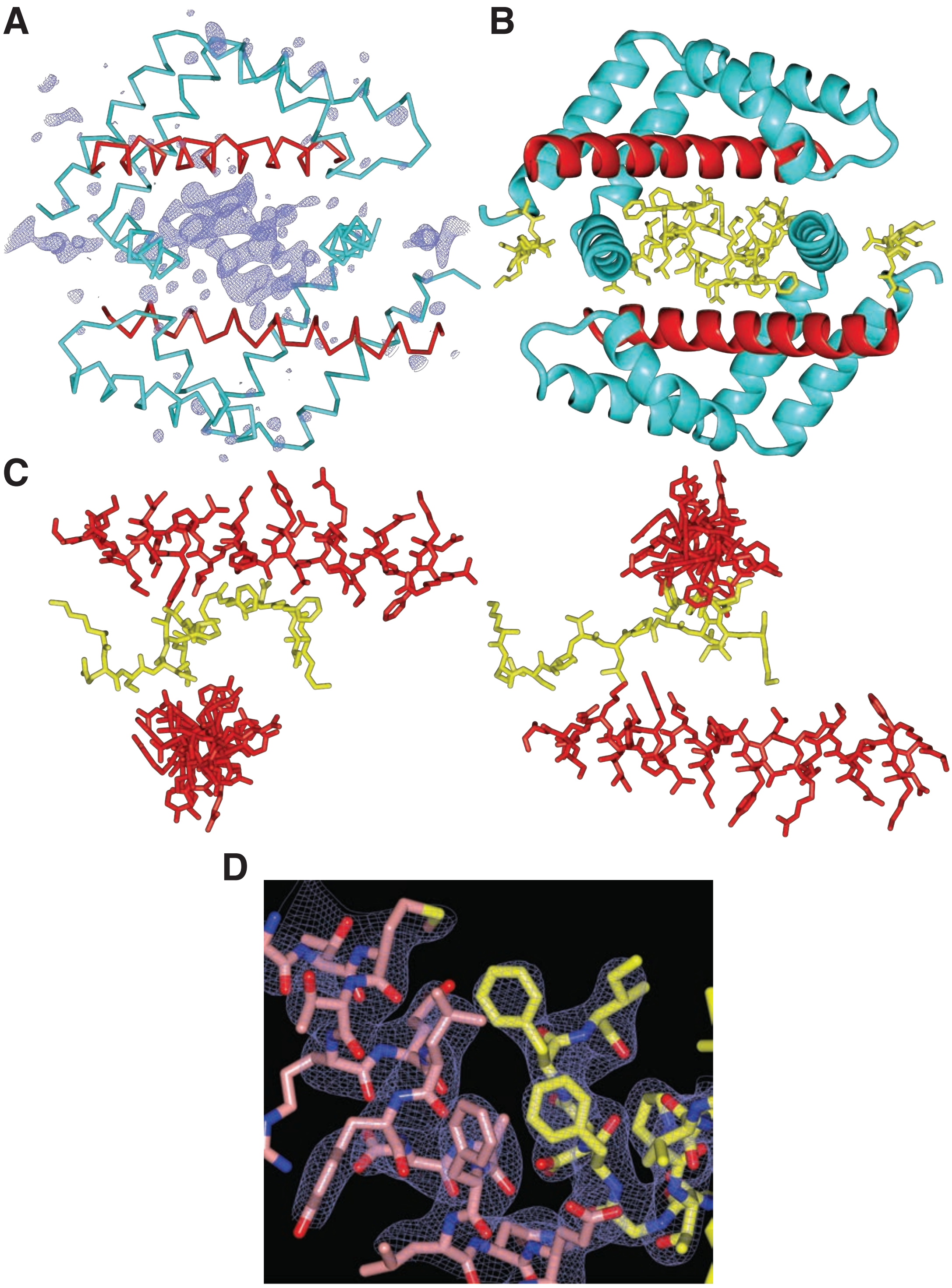

Supplementary Figure S3: Arrangement of protein chains in the *$P3_221$* crystal asymmetric unit (ASU) (A) *Fo-Fc* simulated annealed omit map showing the density of the Nup1 fragment together with the Cα trace for the two Sac3 (red) and Sus1B (cyan) chains. **(B)** The corresponding atomic model of the ASU. **(C)** Two views rotated by 90° about the vertical showing how domain swapping results in one Nup1 chain (yellow) binding to two Sac3 chains (red). **(D)** Final *2Fo-Fc* map showing the primary interface between Nup1 and Sac3.

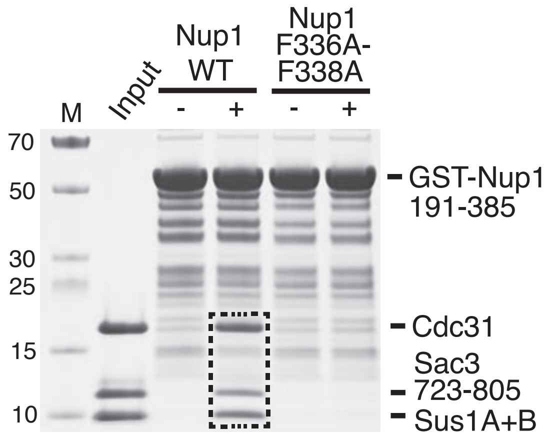

Supplementary Figure S4: Mutation of Nup1 Phe336 and Phe338 abolishes binding to the Sac3^{CID} domain complex Wild-type (WT) and the F336A-F338A mutant of GST-Nup1$^{191\text{-}385}$ were immobilised on glutathione sepharose resin and washed, after which purified Sac3$^{723\text{-}805}$:Cdc31:Sus1 complex (input lane) was added and incubated. Bound material remaining after resin washing, SDS-PAGE and Coomassie staining is shown boxed. M; molecular weight markers (kDa).

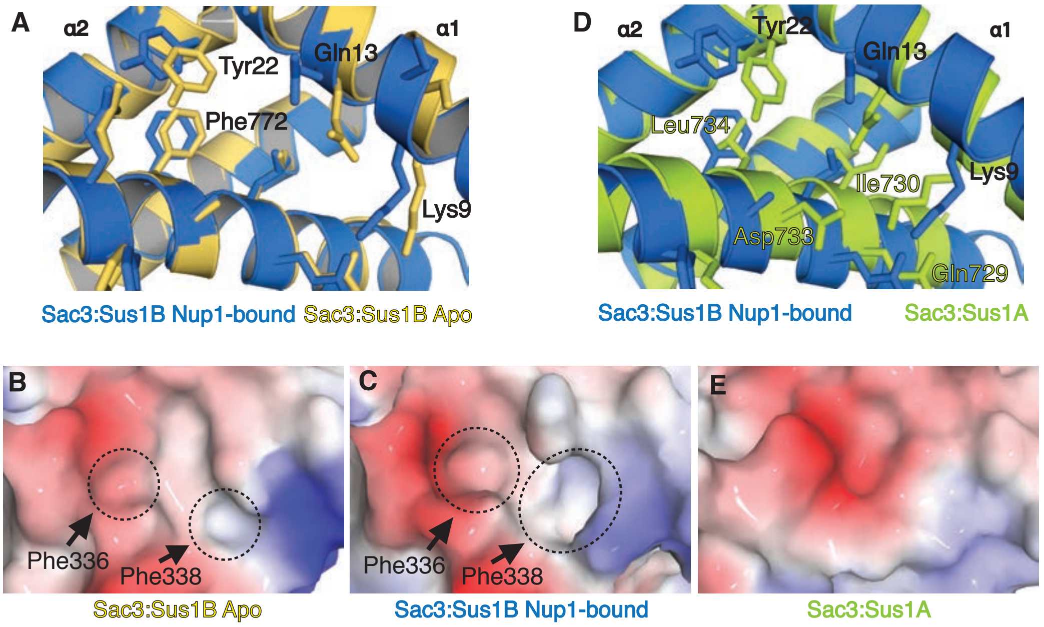

Supplementary Figure S5: Comparison of the binding interface formed by Sac3$^{757\text{-}787}$:Sus1B in apo- and Nup1-bound states and the equivalent interface in Sac3$^{723\text{-}756}$:Sus1A (A) Structural alignment of Sac3:Sus1B from pdb 3FWB (apo, shown in yellow) and Sac3:Sus1B with Nup1-bound (shown in blue) with residues that become buried in the Nup1 interface shown as sticks. Residues whose side-chains undergo rotameric change upon Nup1 binding are labelled. **(B,C)** Electrostatic surface potential of the Sac3:Sus1B interface in the absence and presence of Nup1. The clefts in which Phe336^{Nup1} and Phe338^{Nup1} become buried are marked. **(D)** Structural alignment of Sac3:Sus1A from pdb 3FWC (shown in light green) and Nup1-bound Sac3:Sus1B (shown in blue) with equivalent residues in the structures of Sac3:Sus1A and Sac3:Sus1B shown as sticks and Sac3 residues in the Sac3:Sus1A interface (outline typeface) and those from Sus1A/B (filled typeface) labelled. **(E)** Electrostatic surface representation of the Sac3:Sus1A interface.

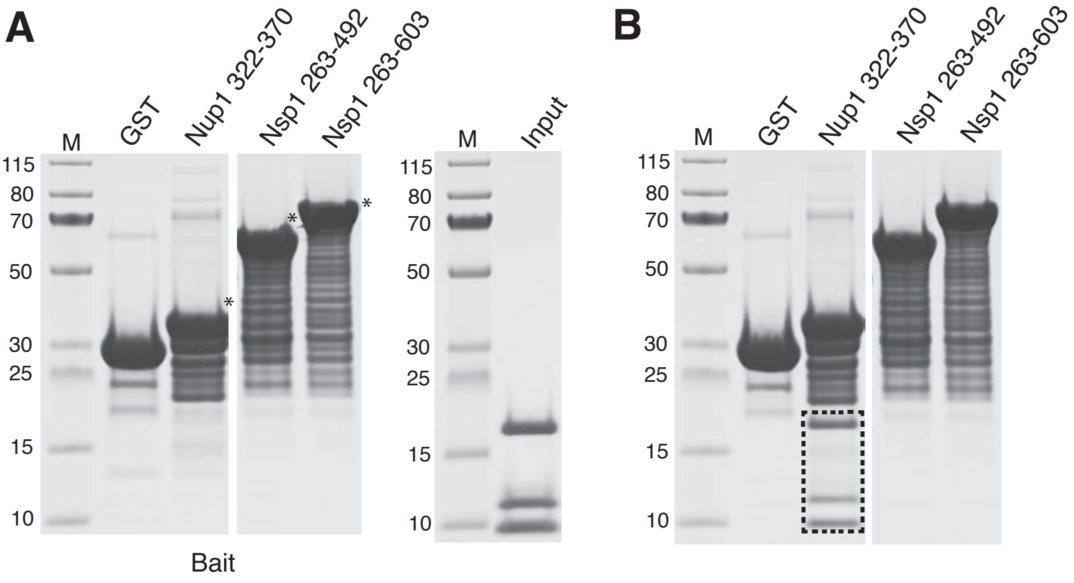

Supplementary Figure S6: The Sac3^{CID} domain complex does not bind to the numerous FxFG repeats within Nsp1 (A) GST, GST-Nup1$^{322\text{-}370}$ and GST-Nsp1$^{263\text{-}492/263\text{-}603}$ were immobilised on glutathione sepharose resin as bait for pull-down assays with purified Sac3^{CID} domain complex used as input. Full-length GST-Nup material is marked by an asterisk. **(B)** Bound material remaining after incubation, resin washing, SDS-PAGE and Coomassie staining is shown boxed. M; molecular weight markers (kDa).

Table S1: Yeast strains used in this study.

Strain	Genotype	Reference
YGC242	*MAT*α *his3*Δ*0 leu2*Δ*0 ura3-*Δ*851 ade2-801 lys2-*Δ*202*::*GFP*::*TETR-LYS2 nup49*Δ::*HIS5MX6* inter*GAL1-FUR4*::(*tetO**112)-*NAT* + pASZ11-NupNop (*ADE2 GFP-NUP49 mCherry-NOP1*)	Berger *et al.*, (2008) *Nature Methods*, **5**, 1031-1037.
DJ43	YGC242 + *SAC3*-mTurquoise2::*URA3*	This study
DJ44	YGC242 + *sac3 L768A H774A*-mTurquoise2::*URA3*	This study
DJ45	YGC242 + *sac3 L768A H774D*-mTurquoise2::*URA3*	This study
DJ46	YGC242 + *sac3 F772A H774A*-mTurquoise2::*URA3*	This study

Table S2: RT-qPCR of *SAC3* expression levels in yeast strains

Strain	Mean C_T (*ACT1*) ± S.E.M.	Mean C_T (*SAC3*) ± S.E.M.	$\Delta\Delta C_T$ ± S.E.	Ratio*	*p*-value**
DJ43	16.8 ± 0.03	23.4 ± 0.04	-	-	-
DJ44	17.0 ± 0.01	23.5 ± 0.01	0.17 ± 0.06	1.1	0.14
DJ45	16.3 ± 0.03	23.0 ± 0.02	-0.04 ± 0.06	0.97	0.62
DJ46	18.0 ± 0.01	24.8 ± 0.05	-0.13 ± 0.08	0.91	0.24

*Calculated quotient between the ratio of the *SAC3* transcript to the *ACT1* transcript in the indicated strain containing mutant *SAC3* relative to the DJ43 strain, which contains wild-type *SAC3*, using the standard $\Delta\Delta C_T$ equation according to the Pfaffl method (1).

**Probability that the level of *SAC3* transcript in the mutant strains is different to that of the wild-type (DJ43), obtained from an analysis of variance between the means of the ΔC_T values between the strain containing mutant *SAC3* relative to the DJ43 wild-type *SAC3* strain using Student's t-test based on a two-tailed distribution with a two-sample unequal variance.

For RT-qPCR analyses of *SAC3* expression in DJ43-46 strains, cells were grown at 30°C exponentially to OD_{600} = 0.5 in synthetic medium with 2% galactose as carbon source. Total RNA was extracted from washed yeast cells using Trizol using a PureLink RNA Mini kit (Life Technologies) and its integrity verified on a denaturing agarose gel. 2 µg of total RNA was treated with DNAse (Promega) and 500ng of DNAse-treated RNA used for reverse transcription (RT) performed with SuperScript III reverse transcriptase kit (Life Technologies) according to manufacturer's instructions using oligo dT(20) primers provided. Primers for qPCR were designed using default qPCR settings in Primer3Plus software (2). The following primer pairs were used for amplification:
ACT1 EV893 (CTTTCAACGTTCCAGCCTTC) and
EV894 (CCAGCGTAAATTGGAACGAC)
SAC3 EV943 (CAAAAGAAGTCGCAGCAACC) and
EV944 (GTTGAATGGTCTTGGCATCG)
Quantitative PCR was performed with 8 µl of cDNA samples comprising a 5-log dilution series using SYBR Green PCR Master Mix (Life Technologies) on a ViiA7 real-time PCR system (Life Technologies). Specificity of amplification was assessed by agarose gel and melting curve analysis. After comparison of primer efficiencies using standard curves, fold changes in *SAC3* transcript abundance in the three strains containing *sac3* mutations (DJ44, DJ45, DJ46) relative to wild-type (DJ43) were calculated using the Pfaffl method (1) based on two biological replicates with three technical repeats of each with arithmetic mean and standard error of the C_T values used to assess variance.

Supplementary References:

1. Pfaffl, M.W. (2001) A new mathematical model for relative quantification in real-time RT-PCR. *Nucleic Acids Res.* **29**:e45.

2. Untergasser, A., Nijveen, H., Rao, X., Bisseling, T., Geurts, R. and Leunissen, J.A. (2007) Primer3Plus, an enhanced web interface to Primer3. *Nucleic Acids Res.* **35**:W71-74.
